# Atypical osteochondroma of the hamate that presented clinically as carpal tunnel syndrome: report of an extremely rare case and literature review

**DOI:** 10.1186/s12891-020-03272-8

**Published:** 2020-04-13

**Authors:** Makoto Motomiya, Taiki Sakazaki, Norimasa Iwasaki

**Affiliations:** 1 Department of Orthopaedic Surgery, Obihiro Kosei Hospital Hand Center|, Obihiro, 080-0024 Japan; 2 Department of Orthopaedic Surgery, Faculty of Medicine and Graduate School of Medicine, Hokkaido University, Sapporo, 060-8638 Japan

**Keywords:** Case report, Carpal osteochondroma, Hamate, Carpal tunnel syndrome, Multilobed, Flexor tendon laceration, Elderly patient

## Abstract

**Background:**

Osteochondroma is a benign tumor that occurs mainly at the metaphysis of long bones and seldom arises from carpal bones. We describe an extremely rare case of osteochondroma of the hamate without a typical cartilaginous cap and with a spiky bony protrusion in an elderly patient.

**Case presentation:**

A 78-year-old right-handed female housekeeper had a multilobed osteochondroma of the hamate, which caused carpal tunnel syndrome and irritation of the flexor tendons. Radiological examinations showed a morphological abnormality of the hamate comprising a spiky bony protrusion into the carpal tunnel and a free body proximal to the pisiform. Open carpal tunnel release and resection of the spiky bony protrusion on the hook of the hamate were performed. The flexor digitorum profundus tendons of the ring and little fingers displayed synovitis and partial laceration in the carpal tunnel. Histological examination also showed atypical findings: only a few regions of cartilaginous tissue were seen in the spiky bony protrusion, whereas the free body proximal to the pisiform contained thick cartilaginous tissue such as a cartilaginous cap typical of osteochondroma. We speculated that the bony protrusion to the carpal tunnel had been eroded by mechanical irritation caused by gliding of the flexor tendon and had resulted in the protruding spiky shape with less cartilaginous tissue. The fractured cartilaginous cap had moved into the cavity within the carpal tunnel proximal to the pisiform and had become a large free body.

**Conclusions:**

Osteochondroma of the carpal bone may take various shapes because the carpal bone is surrounded by neighboring bones and tight ligaments, which can restrict tumor growth. This type of tumor is likely to present with various symptoms because of the close proximity of important structures including nerves, tendons, and joints. The diagnosis of osteochondroma of the carpal bone may be difficult because of its rarity and atypical radiological and histological findings, such as the lack of a round cartilaginous cap. We suggest that surgeons should have a detailed understanding of this condition and should make a definitive diagnosis based on the overall findings.

## Background

Osteochondroma is a benign tumor that occurs mainly at the metaphysis of long bones and is characterized by a round protrusion with a cartilaginous cap [1]. Osteochondroma of the carpal bone is extremely rare; most cases reported involved the scaphoid [2, 3], and only two cases involving the hamate have been reported [4, 5].

Here, we describe an extremely rare case of osteochondroma of the hamate without a typical cartilaginous cap and with a spiky bony protrusion, in an elderly patient.

## Case presentation

A 78-year-old right-handed female housekeeper presented to our department with pain extending from the palm to the forearm during movement of the ring and little fingers, and numbness of the median nerve-innervated area of the left hand. She had experienced intermittent pain and numbness in the hand a few years previously, and the symptoms had gradually worsened starting 1 month before her visit to our department. She had no specific medical and family history except for hypertension.

**Fig. 1:**
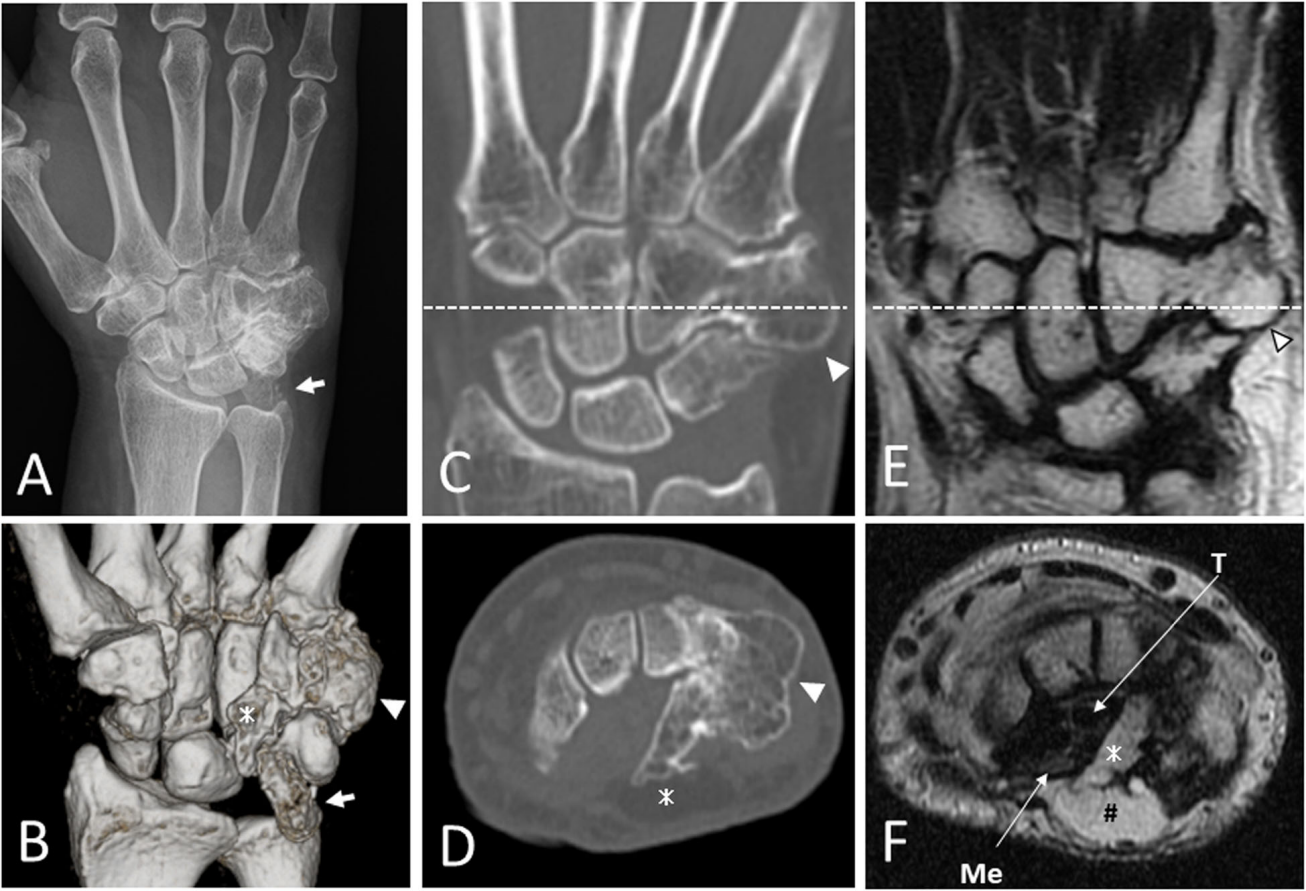
**a** Plain radiographs of the left hand at the first examination showed a morphological abnormality of the hamate and free body proximal to the pisiform (arrow). **b–d** Computed tomography showed a spiky bony protrusion (asterisk) into the carpal tunnel and rounded bony protrusion (arrowhead) on the dorsal–ulnar side of the hamate. **e**, **f** Magnetic resonance images showed that the bony protrusions were connected to the hamate body and had the same intensity as bone marrow. **f** Tenosynovitis of the flexor tendons (T) and edematous changes in the median nerve (Me) were confirmed. The subcutaneous lipoma (hash mark) with high intensity superficial to the spiky bony protrusion was found by chance

On first examination, there was tenderness along the flexor tendons of the ring and little fingers, and the range of motion of the fingers was slightly restricted because of pain. The patient had almost normal sensation in the Semmes–Weinstein monofilament test, although Tinel’s sign was positive at the entrance of the carpal tunnel. Plain radiographs and computed tomography showed a morphological abnormality of the hamate and a free body proximal to the pisiform (Fig. 1a–d). The hamate had a multilobed shape with a spiky bony protrusion into the carpal tunnel and rounded bony protrusion on the dorsal–ulnar side. Magnetic resonance imaging showed that the bony protrusions were connected to the hamate body and had the same intensity as bone marrow (Fig. 1e,f).

**Fig. 2:**
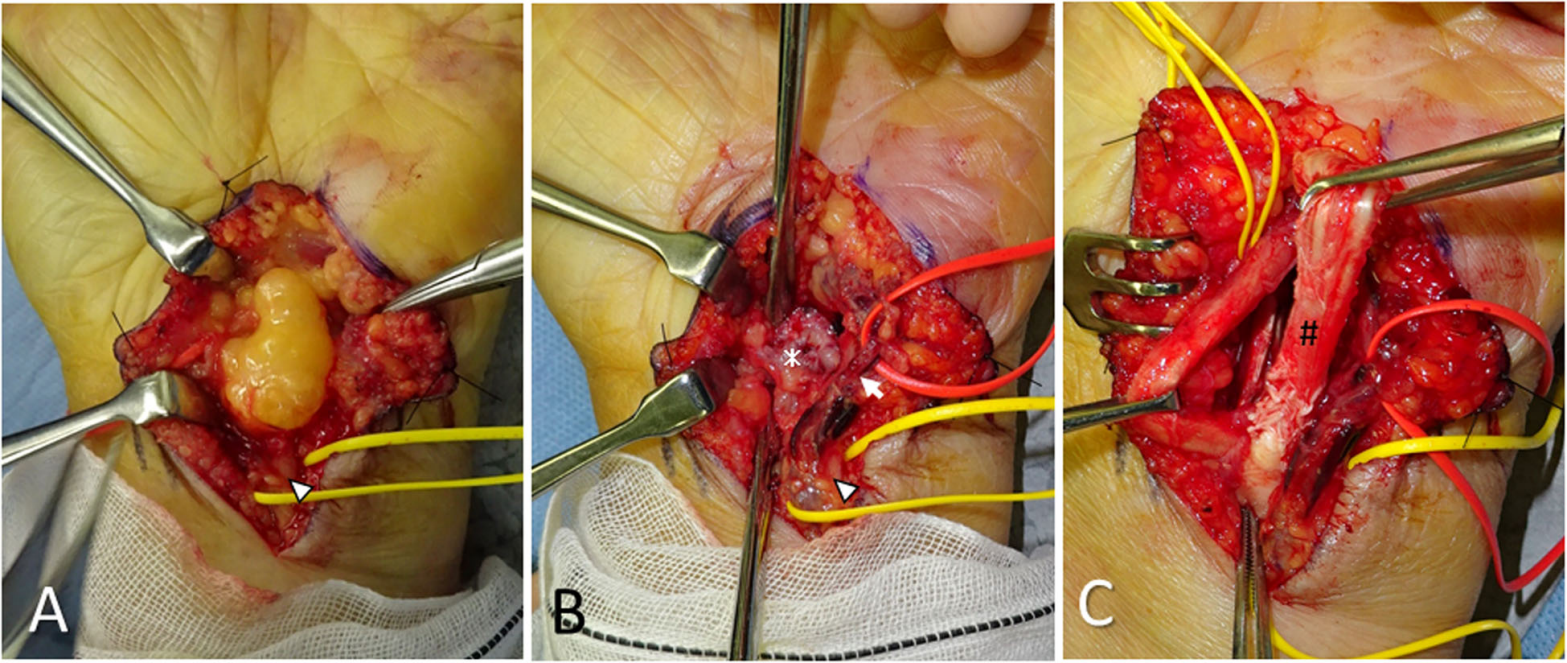
Intraoperative findings. **a** The subcutaneous lipoma was found on the flexor retinaculum and was resected. **b** Open carpal tunnel release and resection of the spiky bony protrusion (asterisk) on the hook of hamate were performed after retracting the ulnar artery (arrow) and the ulnar nerve (arrowhead). **c** The flexor digitorum profundus tendons of the ring and little fingers (hash mark) displayed synovitis and partial laceration in the carpal tunnel

We performed an open carpal tunnel release and resection of the spiky bony protrusion on the hook of the hamate with the patient under regional brachial plexus anesthesia (Fig. 2a, b). The free body proximal to the pisiform was also resected. The flexor digitorum profundus tendons of the ring and little fingers displayed synovitis and partial laceration in the carpal tunnel (Fig. 2c). After performing partial debridement of the lacerated tendons, we confirmed smooth flexor tendon gliding without irritation at the bony protrusion.

**Fig. 3:**
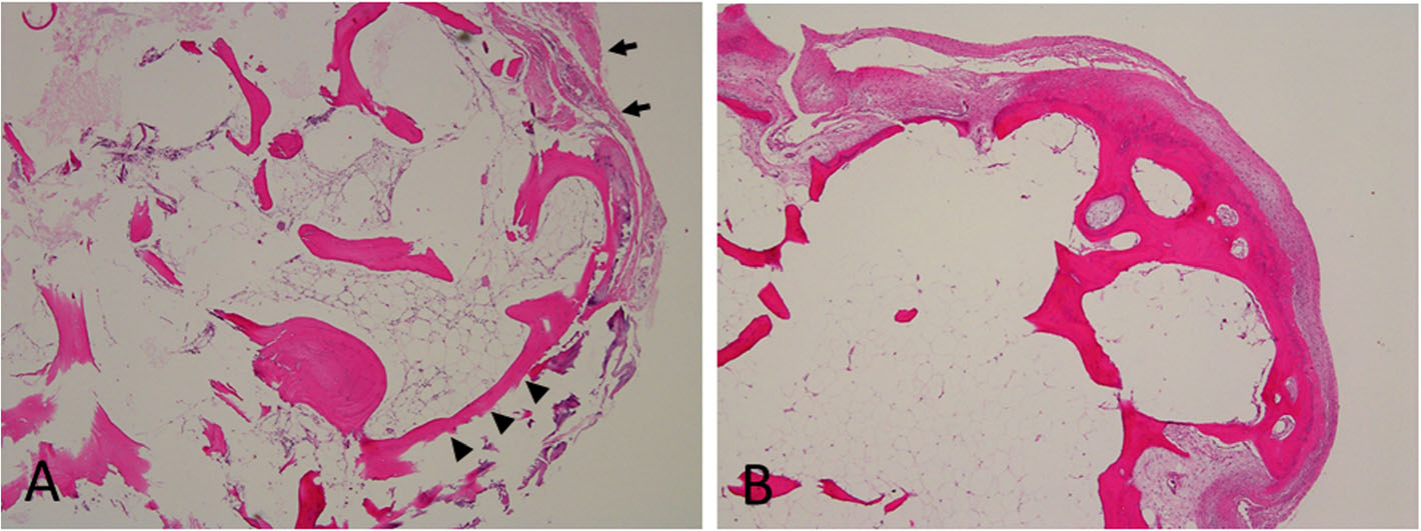
**a** Histological images of the spiky bony protrusion showed only a few regions of cartilaginous tissue (arrows) and no hyperostotic change in the cortex (arrowheads) as in a common osteophyte. **b** Histological images of the free body proximal to the pisiform showed thick cartilaginous tissue such as the typical cartilaginous cap in the common osteochondroma. (Hematoxylin–eosin, × 40)

Histological examination showed only a few regions of cartilaginous tissue in the spiky bony protrusion (Fig. 3a), whereas the free body proximal to the pisiform contained thick cartilaginous tissue such as a cartilaginous cap typical of osteochondroma (Fig. 3b).

**Fig. 4:**
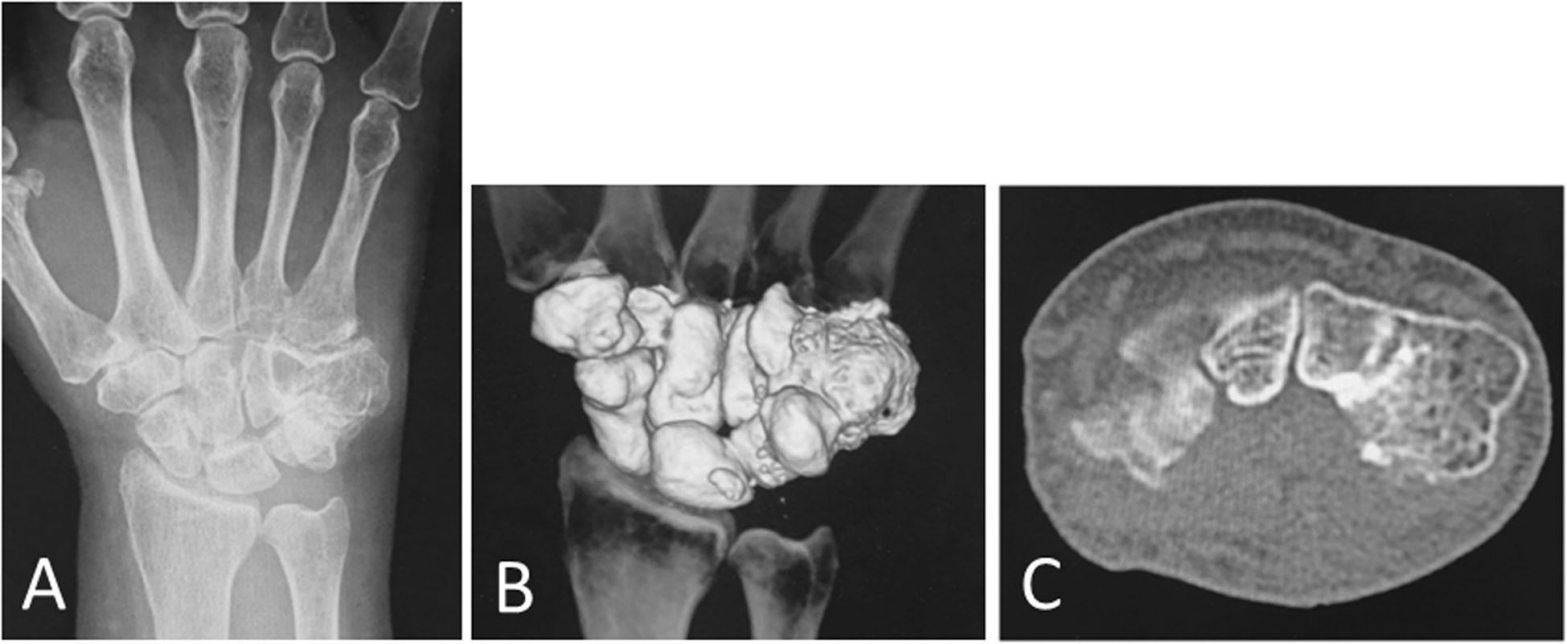
Plain radiograph (**a**) and computed tomography images (**b**, **c**) 6 months after surgery

Six months after surgery, the patient displayed smooth motion of the fingers without pain and numbness. Plain radiographs and computed tomography showed no recurrence of the tumor at the resection site (Fig. 4a–c).

## Discussion and conclusion

Osteochondroma generally originates from the metaphysis of longitudinally growing bones as the site of defects in the periosteum and/or tendon insertion [1]. Because carpal bones have a small surface of periosteum and develop from centrifugally expanding centers of ossification, osteochondroma seldom arises from carpal bones [3]. Osteochondroma in the hamate is extremely rare, and only two cases have been reported [4, 5]. Carpal osteochondroma may take various shapes because the carpal bone is surrounded by neighboring bone and tight ligaments, which can restrict tumor growth [6].

Because the flexor retinaculum originates from the hook of the hamate, osteochondroma of the hook of the hamate can be limited by the linear growth of the flexor retinaculum and can be divided by the retinaculum, which results in a multilobed shape [5]. Of the osteochondromas noted in two previous reports, one was multilobed and the other was unilobed. These osteochondromas spread out in the dorsal–ulnar and volar–ulnar direction outside the flexor retinaculum because of mechanical weakness and caused slight restriction of the wrist range of motion. In our patient, the multilobed osteochondroma had spread both in the dorsal–ulnar direction and within the flexor retinaculum. To our knowledge, this is the first report of a multilobed osteochondroma in the hamate that had expanded into the carpal tunnel and caused carpal tunnel syndrome and irritation of the flexor tendons.

Osteochondroma first appears during the growth period. Symptoms related to the tumor have been reported in patients aged from their teens to around 50 years who seem to use their hand and fingers excessively [1]. A cartilaginous cap protruding into tendons and joints can be exposed to various kinds of mechanical forces such as tendon gliding and/or joint movement, which can lead to fracture and/or deformation [7]. We speculate that the bony protrusion to the carpal tunnel in our patient had been eroded by mechanical irritation caused by gliding of the flexor tendon, which had resulted in the fracture of the cartilaginous cap and the protruding spiky shape with less cartilaginous tissue. In addition, the fractured cartilaginous cap had moved into the cavity proximal to the pisiform and had become a large free body with thick cartilaginous tissue. By contrast, the protrusion in the dorsal–ulnar direction appeared not to have been subjected to mechanical force and had maintained a round shape. Although carpal osteochondroma has not been reported in elderly patients, it is possible that carpal osteochondromas arise because of the long-term effects of mechanical force from surrounding tissues and may show atypical forms in radiographic examination.

Osteochondroma is generally a slow growing tumor that has no pain and is treated conservatively in patients without symptoms [1, 8]. On the other hand, osteochondroma of the carpal bone is likely to present with various symptoms, including restriction of the range of wrist motion, carpal tunnel syndrome, tendon irritation, and/or tendon rupture because of the close proximity of important structures [6, 9]. For osteochondromas presenting with any type of symptoms, the clinician should identify and resect the responsible lesion as soon as possible. Osteochondromas can develop malignant transformation [1, 3], although the malignant transformation of carpal osteochondromas has not been reported. Even if initially asymptomatic, osteochondromas of the carpal bone should be examined carefully and followed up regularly because delayed symptoms can appear as a result of long-term irritation to surrounding tissues.

Given the rarity of carpal bone tumors, the diagnosis of osteochondroma of the carpal bone can be difficult [1, 10]. Because there are bony protrusions in the palm such as the hook of hamate, it is difficult to identify a mass and/or deformity of carpal bone by palpation through the thick soft tissue of the palm. In addition, it may be difficult for radiologists and pathologists to diagnose this condition correctly, especially for cases involving atypical radiological and histological findings such as the lack of a round cartilaginous cap. We suggest that surgeons should have a detailed understanding of this condition and should make a definitive diagnosis based on the overall findings.

In summary, we report a rare case of osteochondroma of the hamate with a spiky bony protrusion and without a typical cartilaginous cap because of mechanical erosion caused by gliding of the flexor tendon. An osteochondroma of the carpal bone may take various shapes because the carpal bone is surrounded by neighboring bones and tight ligaments, which can restrict tumor growth. This type of tumor is likely to present with various symptoms because of the close proximity of important structures including nerves, tendons, and joints. The diagnosis of osteochondroma of the carpal bone may be difficult because of its rarity and atypical radiological and histological findings, such as the lack of a round cartilaginous cap. We suggest that surgeons should have a detailed understanding of this condition and should make a definitive diagnosis based on the overall findings.

## Data Availability

This is a case report of a single patient. To protect privacy and respect confidentially, no raw data have been made available in any public repository. The original operation reports, imaging studies, and outpatient clinic record are retained as per normal procedure within the medical records of our institution.
